# The affective neuroscience of socioeconomic status: implications for mental health

**DOI:** 10.1192/bjb.2020.69

**Published:** 2020-10

**Authors:** Yu Hao, Martha J. Farah

**Affiliations:** 1 Center for Neuroscience & Society, University of Pennsylvania, USA; 2 Center for Neuroscience & Society, University of Pennsylvania, USA

**Keywords:** Socioeconomic status, mental health, fMRI, emotion, emotion regulation

## Abstract

We review basic science research on neural mechanisms underlying emotional processing in individuals of differing socioeconomic status (SES). We summarise SES differences in response to positive and negative stimuli in limbic and cortical regions associated with emotion and emotion regulation. We discuss the possible relevance of neuroscience to understanding the link between mental health and SES. We hope to provide insights into future neuroscience research on the etiology and pathophysiology of mental disorders relating to SES.

Low socioeconomic status (SES) is a potent risk factor for mental disorders, particularly mood and anxiety disorders.^1–3^ Life's challenges weigh more heavily on people with fewer financial resources and less education, and the impact of SES on emotional well-being is substantial. For example, Guhn et al^4^ found an adjusted odds ratio for mental health conditions 25–39% higher for children of low-income families compared with others. A Canadian government survey^5^ found that disabling mental health problems are twice as common among those without a high school diploma compared with college graduates.

In this article we review what is known about the affective neuroscience of SES and sketch some of the implications this field of science might have for mental health. We begin with basic definitions and distinctions. ‘Affective neuroscience’ is the study of neural mechanisms underlying the experience, expression and regulation of emotion. Given that the brain is shaped by a combination of genes and environment, explanations in terms of the brain and the environment are not mutually exclusive. SES is a complex construct, or more accurately an interrelated set of constructs, that captures differences in material and social wherewithal. It has been measured in terms of financial attributes (e.g. income, wealth), neighbourhood characteristics (e.g. rates of unemployment and crime near one's home), educational and occupational background and self-reported social standing, among other approaches. Although these measures are all distinct from one another,^6^ they are normally moderately correlated.^7^ Here we will seek broad generalisations within this relatively small literature, referring therefore to SES in general rather than making distinctions among more specific measures. Similarly, we will generalise across life stages at which participants’ SES was measured. All of these distinctions undoubtedly have scientific and clinical relevance, which should also be examined. Nevertheless, for a preliminary review, we choose to begin by aggregating findings as much as possible.

Many useful insights into the aetiology and pathophysiology of mental disorders have come by studying the brain. Here we explore the possible relevance of neuroscience to understanding the link between mental health and SES. Recent attempts to link SES and psychopathology through the brain have adopted different concepts from psychology and neuroscience as explanatory frameworks. These include the effects of stress on the brain,^8^ the role of self-regulation in mental health^9^ and the distinction between adversities consisting primarily of deprivation (prevalent in low SES) and those consisting primarily of threat (such as accompanies abuse^10^). These frameworks are not mutually exclusive, and the goal of our review is neither to adjudicate between them nor to develop a fourth alternative. Rather, we aim to gather the most comprehensive collection to date of research findings related to SES, brain and affect, and attempt a provisional integration that will enable empirical generalisations and highlight consistencies and inconsistencies.

The neuroscience studies reviewed here are basic science research, carried out with normal people of varying SES. Note that in some studies SES is not the focus of the study but included as a covariate. In some of these cases the range of SES is restricted and the power to detect SES effects is therefore attenuated. Figure 1 depicts the systematic review process.

**Fig. 1:**
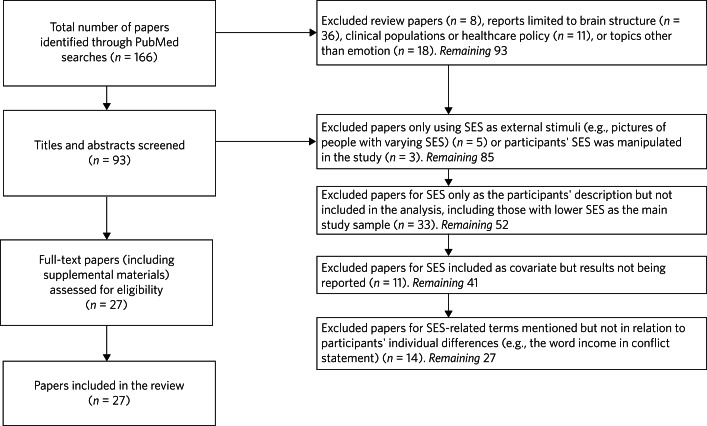
Flowchart for the systematic review. Relevant studies were identified through searches of the database PubMed throughout 13 March 2020. The search required that studies used at least one of the following socioeconomic status (SES) keywords in the full paper: socioeconomic status, poverty, income, neighbourhood quality, neighbourhood disadvantage, educational attainment, social class, social standing. Identified studies also used at least one of the following social and affect-related keywords in the entire paper: emotion, threat, fear, angry, sad, happy, reward, social interaction, hostility, rejection. In addition, the keyword of neuroimaging method was included: fMRI. This search identified 166 results, which were screened for the inclusion criteria.

The resulting functional magnetic resonance imaging (fMRI) studies manipulated emotional states experimentally inside the scanner with emotionally evocative stimuli or tasks. The most common methods involve photographs of faces expressing different emotions or of affectively valenced objects and scenes, and signals that money has been won or lost in a game. Less frequently, studies have used other kinds of stimuli or experiences to evoke emotion and these will also be described.

## Subcortical and cortical bases of negative affect

Textbook coverage of affective neuroscience invariably begins with subcortical systems, particularly the amygdala and ventral striatum. These appear to respond automatically, without the need for conscious awareness of the emotion (e.g.^11,12^), and play crucial roles in the experience and expression of emotion. Many other regions, cortical and subcortical, also participate in emotion and there is no simple mapping of specific emotions to specific brain areas.^13^ Emotion is best thought of as a construction of the brain as a whole.^14^ Nevertheless, many studies take the amygdala as a starting point in the investigation of emotion and the brain, especially negative emotion.

### Brain responses to negative facial emotions

Although the amygdala plays a role in a range of emotions, its most consistent role is in negative emotions. The amygdala is most readily activated by viewing facial expressions of fear and anger. Our review identified a substantial number of studies that have compared low- and high-SES individuals under these experimental conditions. In the majority of studies, lower SES by at least one measure was associated with stronger amygdala reactivity to negative facial emotions. Young adults whose parents were of lower SES showed greater amygdala activation when viewing angry faces compared with control conditions in two studies.^15,16^ In another study, negative facial expressions elicited greater amygdala reactivity for young adults from lower-SES families, taking into account parenting quality and maternal mental health.^17^ Emotional faces more generally evoked greater right amygdala activation in adults of lower SES.^18^ The same study showed that SES was associated with strength of coupling between right amygdala and right insula.^18^ In a study of first-time mothers viewing the faces of either happy or distressed infants, the right amygdala was more active to depictions of distress in mothers of lower SES.^19^

Three other studies yielded partial support for a relationship between amygdala reactivity to negative facial emotions and SES. The support from these findings was qualified by participant gender in one case (only for women^20^), ethnicity of depicted face in another (only for Black emotional faces in a study with Mexican American participants^21^) and history of violence exposure in a third (only in non-exposed participants^22^). In only one study testing the relationship between amygdala reactivity and SES was a significant relationship not found, and this study had relatively little variance in SES among the participants (all were recruited through an antipoverty programme^23^). Finally, in a memory study with emotional faces as retrieval cues, testing the hippocampus as a region of interest (ROI), there was less activity in the posterior hippocampus in lower-SES individuals when they watched angry faces.^24^

### Brain responses to other negative stimuli

Apart from facial emotional cues, other stimuli that have been used to evoke negative emotions include unpleasant sounds, social rejection and loss of money. In one study unpleasantly loud auditory stimuli were used to evoke emotional states in participants of varying SES, violence exposure and ethnicity, generally with a warning tone but occasionally without.^25^ When trials without a warning cue were considered, family income was negatively associated with hippocampal activity, consistent with greater effect of unexpected negative stimuli with lower SES. SES showed the opposite effect with cued noise, with higher neighbourhood deprivation (i.e. lower SES) showing lower activity in the hippocampi and amygdalae, perhaps related to reduced preparatory regulatory effects.

Two studies have used the sound of babies crying, contrasted with white noise, with participants of varying SES. The crying was rated as more annoying,^26^ but effect of SES on neural response to the cry in non-parent adults was complex and difficult to interpret; there was generally more activation in the insula and several other regions in women from low-SES backgrounds, with the opposite pattern in men. Another study of mothers hearing baby cries found less activation associated with lower SES in frontal and temporal cortical regions, but differences in classic emotion-related areas were not found.^27^ Although baby cries communicate urgency and distress, they may function less as generic signals of threat or harm and more as triggers for complex, evolved systems of parenting behaviour.^28^

Social rejection is another kind of experience that evokes negative emotion. The so-called ‘social pain’ that results from rejection is not typically associated with amygdala activity, but has a more distinctive functional anatomy including the dorsal and ventral anterior cingulate, anterior insula and also the ventromedial prefrontal cortex (vmPFC) and orbital cortex regions (see meta-analyses^29–31^). Gonzalez et al^32^ assessed responses to exclusion in the classic ‘cyberball’ rejection task in young adults of varying neighbourhood SES, and tested whether SES was associated with dorsal anterior cingulate cortex (dACC) and insula responses. The insula was not reliably activated by exclusion in this study overall, nor as a function of SES, but lower SES was associated with a larger dACC response. Related to social rejection is negative social evaluation. Muscatell et al^33^ had participants read negative versus neutral or positive personal assessments of their performance in an interview, and *a priori* regions of interest were the dorsomedial prefrontal cortex (dmPFC) and amygdala. Although amygdala activity did not differ with subjective social status (and it was not stated whether the task evoked amygdala activity in this condition for participants in general), dmPFC activity was evoked by negative evaluations and was higher in lower-SES participants.

Finally, the aversive experience of losing money, a secondary reinforcer, which differs in some ways from primary aversive stimuli such as pain,^34^ has been assessed during fMRI in two studies with participants of varying SES, neither of which found effects of SES.^35,36^

### Summary

There is a trend, across various forms of negative emotional state, for lower-SES individuals to have more brain activity in at least some emotion-related areas. This includes evidence from tasks using emotional facial expressions, loud noises and social rejection. This generalisation, although reasonably broad, does not extend to all of the literature. In particular, two studies using infant cry sounds show SES effects, but these effects are complex and cannot be interpreted as simply more activity in emotion-related areas, and two studies of monetary loss failed to show effects of SES at all.

## Subcortical and cortical bases of positive affect

The anatomy of positive affect overlaps with some of the areas mentioned above in connection with negative affect, consistent with the complex, emergent nature of emotion in the brain. Studies of positive affect use depictions of happy faces and scenes and the occurrence of desirable outcomes such as the winning of money or points in games. The region most often associated with positive affect is the ventral striatum, which consists primarily of the nucleus accumbens and part of the caudate nucleus, although other cortical regions are also engaged, including the medial and orbital frontal cortex, cingulate cortex and anterior insula.^37^

The literature on the neural correlates of positive emotion and SES is relatively small. One study, already mentioned in connection with negative emotion, is also relevant to positive emotion. In this study, mothers viewed happy as well as unhappy baby faces, and some of the areas activated by the happy faces, including the left amygdala and the right insula, differed by SES and specifically were less active in the lower-SES mothers.^19^

Silverman et al^38^ exposed participants to affectively valenced pictures of people, objects and scenes and contrasted neural responses to positive images (e.g. an amusement park) relative to neutral images (e.g. furniture). They found lower activity in response to the happy pictures in lower-SES participants in a variety of areas, including the striatum.

Other studies have induced positive emotional states with monetary gains during simple games. The focus of most studies on SES and reward has been on reward anticipation, rather than the receipt of the reward itself. Reward anticipation is a motivational state sometimes associated with ‘wanting’, in contrast to the response of ‘liking’.^39^ Response to receipt of a reward has either not been shown to differ by SES^35,40^ or the study design has not allowed reward receipt to be examined separate from reward anticipation because of block rather than event-related design.^36^ In contrast, reward anticipation generally evokes more activity for lower-SES participants. Romens et al^40^ found heightened response to the anticipation of reward in medial prefrontal cortex (mPFC) in lower-SES girls, and no locations of reduced response. Gonzalez et al^41^ found a similar relationship in striatal and other regions. Quevedo et al^35^ covaried SES in a study of the effect of attachment style on reward, and although the range of SES was relatively narrow, they found that maternal unemployment and lower family income during childhood were associated with higher striatal activity and amygdala activity respectively, during reward anticipation. When anticipating a larger but lower-probability reward, lower-SES adolescents show more mPFC activation.^42^

### Summary

Positive stimuli may evoke smaller responses in people of lower SES, although the evidence is limited, while reward anticipation may be accompanied by greater activation.

## Networks for emotion and emotion regulation

Emotion regulation refers to self-induced changes in intensity and duration of emotional experience, typically for the purpose of reducing negative experience. These changes can be accomplished by either conscious, explicit strategies or automatic, implicit processes.^43^ One of the most effective explicit emotion regulation strategies is cognitive reappraisal, by which we volitionally reinterpret the meaning of stimuli in order to alleviate negative feelings. A recent review suggests that explicit emotion regulation engages the dorsolateral, ventrolateral and dorsomedial frontal and parietal cortex.^44^ Kim et al^45^ showed disturbing pictures to participants of varying SES and instructed them to reduce negative emotion through cognitive reappraisal, for example viewing a picture of a bruised and beaten woman and reappraising it as a picture of an actress playing the role of a violence victim. They found that individuals of low SES recruited less prefrontal activation than their higher-SES counterparts and showed less reduction in amygdala activity during reappraisal, consistent with this emotion regulation strategy being used less effectively by these participants. However, gender seems to moderate the effect of SES on prefrontal activity related to emotion regulation:^20^ in males but not females when considered separately, activation in dorsolateral and ventrolateral prefrontal cortical regions (dlPFC and vlPFC) during cognitive reappraisal was positively correlated with SES.

Another form of emotion regulation is implicit, involuntary emotion regulation, which does not require effortful use of a strategy or conscious monitoring of emotional state, but is simply evoked automatically.^46^ Implicit emotion regulation is omnipresent in our encounters with emotional stimuli, with ventral ACC and vmPFC engaging automatically to modulate subcortical activity.^44^ By its nature, implicit regulation is not carried out following instructions, so it can be difficult to determine in any given task whether these ventral anterior activations represent regulatory activity. In any case, less functional coupling between the amygdala and vmPFC has been found in low-SES individuals when processing negative emotion.^47^ In the same intensively studied group of participants (see also^20,45,47^), Liberzon and colleagues^48^ found less prefrontal activity in lower-SES participants in a task designed to evoke implicit emotion regulation, although in this task the finding was localised to lateral rather than medial regions.

Studies of participants at rest provide additional evidence concerning limbic–cortical interactions. Functional connectivity between the amygdala and the vmPFC was found to be weaker in participants of lower SES, and this difference in brain activity accounted for SES disparities in vulnerability to stressful life events.^49^ Connectivity of the amygdala and hippocampus to prefrontal regions was also reduced in lower-SES children, and these differences fully mediated the relationship between SES and later depression.^50^ Finally, connectivity between the ventral striatum and ventral PFC is reduced in low SES, and this fully mediated the relationhip between SES and anxiety.^51^

## Conclusions

We offer this preliminary review of the literature as a starting point, to be refined as the literature grows and our understanding of SES and affective neuroscience advances. It is limited in part by the small size of the literature. Our search method uncovered only 27 studies, and many of these involved small samples (*n* < 50 for half of the studies) or a restricted range of SES. In addition, affect and SES are both complex constructs, and each has been operationalised in numerous different ways in the studies reviewed here. Is it sensible to group the sight of a frightened face, the sound of a crying baby and the loss of small sums of money into a common category of ‘negative emotion?’ We did so here provisionally, to help organise our review at a very general level, recognising that important differences may be glossed over. The studies reported here were also heterogeneous in terms of participants’ ages. Finally, the studies measured SES in different ways, for example in childhood or adulthood, and according to income, educational attainment or neighbourhood deprivation. In attempting this first broad review of SES and the neural bases of emotion, we do not distinguish between different measures of SES, and we report findings as positive if any measure of SES shows a statistically significant effect.

For the reasons just mentioned, any conclusions from this review must be considered very provisional. Nevertheless, some trends can be discerned, and these may be relevant to the SES gradient in mood and anxiety disorders. Socioeconomic disadvantage shapes the brain's response to emotional stimuli, such that negative stimuli appear to evoke a stronger response and positive stimuli may possibly evoke a weaker response. This amounts to an overall bias towards the negative and away from the positive for lower SES, which would be expected to indicate a greater susceptibility among low-SES individuals to depression and anxiety. In contrast, anticipation of reward appears to evoke more activity in people of lower SES, which in one study mediated the relationship between socioeconomic disadvantage and adolescent depression symptoms.^40^ Greater reactivity to the promise of reward may contribute to disorders of impulse control.^52^ Finally, in at least a few studies functional networks that may serve to regulate emotion are weaker in people of lower SES, and these differences too have been found to mediate risk for psychopathology.

Given the disproportionate mental health burden borne by those of low SES, it makes sense to deploy the full range of approaches to understanding and addressing this inequity, from the sociological to the neuroscientific. High priorities for future research will be to expand the evidentiary base relating SES, brain function and psychological symptoms, and to establish how social and economic factors external to the individual may give rise to the neural and psychological vulnerabilities reviewed here. In aiming to understand the interrelations among psychology, biology and social context, it should be possible for mental health and well-being to be more widely enjoyed throughout society.

## About the authors

**Yu Hao**, PhD, is a postdoctoral researcher at the Center for Neuroscience & Society, University of Pennsylvania, Philadelphia, Pennsylvania, USA. **Martha J. Farah**, PhD, is Walter H. Annenberg Professor in the Natural Sciences and Director of the Center for Neuroscience & Society, University of Pennsylvania, Philadelphia, Pennsylvania, USA.

## Supplementary material

For supplementary material accompanying this paper visit https://doi.org/10.1192/bjb.2020.69. click here to view supplementary material
