# The affective neuroscience of socioeconomic status: implications for mental health – CORRIGENDUM

**DOI:** 10.1192/bjb.2021.99

**Published:** 2022-08

**Authors:** Yu Hao, Martha J. Farah

**Keywords:** Socioeconomic status, mental health, fMRI, emotion, emotion regulation

The authors would like to correct a sentence in the above mentioned paper.

In the first paragraph in the second column on page 2, the below paragraph:

in another study, negative facial expressions elicited **greater** amygdala reactivity for young adults from lower-SES families, taking into account parenting quality and maternal mental health.^17^

Should instead read:

in another study, negative facial expressions elicited **less** amygdala reactivity for young adults from lower-SES families, taking into account parenting quality and maternal mental health.^17^

The authors apologises for this error.
